# The Association between Working Posture and Workers’ Depression

**DOI:** 10.3390/healthcare10030477

**Published:** 2022-03-03

**Authors:** Ka Young Kim

**Affiliations:** Department of Nursing, College of Nursing, Gachon University, Incheon 21936, Korea; kykim@gachon.ac.kr

**Keywords:** depression, occupation health, workers, working posture

## Abstract

Various studies have focused on the association between physical health and working posture. However, little research has been conducted on the association between working posture and mental health, despite the importance of workers’ mental health. This study aimed to examine the association between working posture and workers’ depression. A total of 49,877 workers were analyzed using data from the 5th Korean Working Conditions Survey. We utilized multiple logistic regression to analyze the variables associated with workers’ depression. This study showed that several working postures, such as tiring or painful positions, lifting or moving people, standing, and sitting, were associated with depression in workers. Furthermore, occupation types, job satisfaction, and physical health problems related to back pain and pain in upper (neck, shoulder, and arm) and lower (hip, leg, knee, and foot) body parts were associated with workers’ depression. Therefore, this study demonstrated that working posture is associated with workers’ depression. In particular, working postures causing musculoskeletal pain, improper working postures maintained for a long time, and occupation types were associated with workers’ depression. Our findings demonstrate the need for appropriate management and interventions for addressing pain-inducing or improper working postures in the workplace.

## 1. Introduction

According to the WHO, occupational health is an important field for public health to promote and maintain the physical, mental, and social welfare of workers [1]. Occupational health is affected by various working conditions and environments [2,3]. In particular, working posture is closely correlated with workers’ safety and health [4,5]. Poor working posture can cause risk of injury in the industrial workplace [6,7]. Furthermore, industrial workers suffer from musculoskeletal disorders (MSDs) due to improper working postures, including bending, twisting, overreaching, repetitive tasks, and uncomfortable positions [8]. A previous study reported that sand core making workers who grind in awkward positions also suffered from MSD, such as low back, hand, shoulder, wrist, and neck pain [5]. Work-related neck and shoulder pain was found to be related to the work environment, including awkward working posture and prolonged sitting, in self-employed tailors in Ethiopia [7].

As noted above, various studies have focused on the physical health ramifications of working posture [5,7,8]. However, it is also necessary to consider the relationship between working posture and workers’ mental health. Workers’ mental health is an essential issue in the workplace. Depression and anxiety are associated with a significant economic impact as well as workers’ quality of life [9,10]. Environmental factors, such as noise and temperature, are also associated with psychological stress [11]. Reportedly, occupational exposure was associated with depression in the French national working population [12]. Furthermore, working environments, including setup and personal protective equipment availability, were associated with anxiety levels in healthcare workers [13]. The physical working environment, including all the material objects and stimuli that workers are exposed to in the workplace, is known to be associated with mental health. However, to the best of our knowledge, little research has been conducted on the association between working posture and mental health [11].

This study aimed to examine the association between working posture and workers’ depression and to provide a basis for managing and maintaining a work environment that protects workers’ mental health.

## 2. Materials and Methods

### 2.1. Study Design and Participants

This study was a cross-sectional examination aimed at demonstrating the association between workers’ working postures and depression. Data were collected from the 5th Korean Working Conditions Survey (KWCS) conducted by the Korea Occupational Safety and Health Agency, a government-funded institute under the Ministry of Employment and Labor, in 2017 [14]. This KWCS was designed to identify an overall working environment that exposed workers to hazards or to risk factors in varying types of occupation, business, and employment and used multistage random sampling in 17 cities and provinces of the Republic of Korea. The data were household visit interview surveys conducted using Computer Assisted Personal Interviewing, a face-to-face interview method using a tablet PC. A total of 50,205 subjects who were in the economically active population and older than 15 years participated in the survey, including workers, business owners, and self-employed persons. In this study, we included 49,877 workers after excluding 328 workers whose data had missing values.

### 2.2. Study Variables

This KWCS data included socio-demographic variables, work-related health variables including physical and mental, and variables to work-related postures. Socio-demographic variables included age, sex, education, occupation, shift work, and job satisfaction. Occupation was categorized into four groups: (a) Administrator and professional (WC worker). (b) Engineer, semi-professional, and office worker (BC worker). (c) Service and sales worker (PC worker). (d) Agriculture, forestry, and fishery industry skilled worker; technical skills and related skills worker; equipment machinery operator and assembly worker; simple labor worker; and soldier (AL worker). Shift work had two responses: no or yes. Job satisfaction was rated from 1 to 4: 1: not at all satisfied; 2: not very satisfied; 3: satisfied; and 4: very satisfied. Work-related health variables included back, neck, shoulder, and arm pain; hip, leg, knee, and foot pain; and depression. These were assessed by two responses: no or yes. Work-related postures included tiring or painful positions, lifting or moving people, carrying or moving heavy loads, standing, sitting, and repetitive hand or arm movements. These were scored on a scale of 1 to 7 according to the corresponding level at work: 1: never; 2: almost never; 3: around ¼ of the time; 4: around half of the time; 5: around ¾ of the time; 6: almost all the time; and 7: all the time. Finally, the presence of depression was marked by the presence or absence of any depression symptoms due to work over the last 12 months.

### 2.3. Statistical Analysis

Data analyses were conducted using IBM SPSS Statistics (version 26; IBM Corp., Armonk, NY, USA). Descriptive data were presented as mean, standard deviation, frequency, and percentage according to the presence of depression. Pearson’s correlation, ANOVA, and *t*-test were used to analyze the statistical significance. Multiple logistic regression was performed to identify the influence of socio-demographic and work-related variables on workers’ depression. Statistical significance was set as a *p*-value of less than 0.05.

## 3. Results

### 3.1. General Characteristics of Workers

Table 1 shows the general characteristics of workers according to depression. Significant variables related to depression were age; sex; education; occupation; job satisfaction; and physical health problems, including back pain, and pain in specific upper (neck, shoulder, and arm) and lower (hip, leg, knee, and foot) body parts. Significant working postures were tiring or painful positions, carrying or moving heavy loads, standing, and repetitive hand or arm movements.

### 3.2. Working Posture Affecting Workers’ Depression

The logistic regression results show the variables affecting workers’ depression (Table 2). Regarding working posture, the odds ratio of maintaining tiring or painful positions, standing, and sitting was 1.096, 0.938, and 1.049 times higher, respectively, with depression. In males, the odds ratio of maintaining tiring or painful positions and lifting or moving people was 1.098 and 1.101 times higher, respectively, with depression. In females, the odds ratio of maintaining tiring or painful positions, standing, and sitting was 1.095, 0.938, and 1.068 times higher, respectively, with depression.

In occupation, BC (Odds Ratio (OR) = 0.755) and AL workers (OR = 0.549) were less likely to be depressed than WC workers. In males, BC (OR = 0.613), PC (OR = 0.651), and AL workers (OR = 0.393) were less likely to be depressed than WC workers. The odds ratio of shift work was 1.383 times higher only in males. Those with increased job satisfaction were less likely to be depressed in the total (OR = 0.460), male (OR = 0.424), and female (OR = 0.480) samples. Physical health problems which affect posture, back pain, and pain in specific upper (neck, shoulder, and arm) and lower (hip, leg, knee, and foot) body parts were 2.035, 2.164, and 2.344 times more likely, respectively, with depression. In males, back pain and pain in specific upper (neck, shoulder, and arm) and lower (hip, leg, knee, and foot) body parts was 1.879, 2.132, and 2.756 times more likely, respectively, with depression. In females, back pain and pain in upper (neck, shoulder, and arm) and lower (hip, leg, knee, and foot) body parts was 2.130, 2.079, and 2.102 times more likely, with depression.

## 4. Discussion

This study demonstrated the association between working posture and depression in workers.

Working posture is important for the health of workers in the workplace. However, to the best of our knowledge, few studies have shown that specific working postures are related to specific diseases. Furthermore, it is not easy to elucidate the effects of a specific working posture on depression [15]. Depression is a complex illness that has various possible causes, including genetic vulnerability, faulty mood regulation by the brain, stressful life events, and environmental exposure [16,17]. Several studies have demonstrated the association between depression and posture, but not working posture, although it is still difficult to elucidate the cause-and-effect relationships in the association between depression and posture. Depression has been noted to be related to changes in posture, such as increased head flexion, increased thoracic kyphosis, a trend toward pelvic retroversion, and an increase in scapular distance [18]. Furthermore, participants with depression had significantly more slumped posture than the healthy community sample, while postural manipulation significantly improved positive affect and reduced fatigue [19]. Thus, this study demonstrated the association between working posture and workers’ depression after adjustment for socio-demographic and working posture related physical health problem variables that were known to affect workers’ depression.

Interestingly, in this study, several working postures, such as tiring or painful positions, lifting or moving people, standing, and sitting, were associated with depression in workers. A study reported that shoulder pain was the result of risk factors such as physical work with heavy loads, awkward work postures, mental stress, and obesity [20]. Musculoskeletal pain has been reported to be an important factor in depression in the general working population of Denmark [21]. Thus, it seems that tiring or painful positions are involved in depression. Next, the working posture of lifting or moving people was significantly associated with depression among male workers. There is no direct evidence of this; however, it appears that male workers are more commonly exposed to working conditions that involve lifting or moving people than female workers and experience more stress, leading to depression [22]. Furthermore, standing and sitting working postures were negatively and positively associated with depression in workers, respectively. A study reported that office workers who spent a long time in a sedentary position showed increased discomfort and creative problem-solving errors [22]. Furthermore, an upright posture can decrease depression by improving breathing [23]. However, as shown in several studies, it appears that incorrect or poor working postures are more likely to be associated with depression than some specific working postures [24,25].

Moreover, occupation types, job satisfaction, and physical health problems related to back pain; neck, shoulder, and arm pain; and hip, leg, knee, and foot pain were associated with workers’ depression. Job satisfaction is a well-known factor that is associated with decreased depression among workers [26,27]. In regard to occupation type, jobs that use more physical movement and mostly involve standing were associated with less depression than white-collar workers who mostly work at a desk. Finally, similarly to other studies, we found that musculoskeletal pain, specifically pain in the back and the upper and lower body parts, was significantly related to workers’ depression.

However, it is still not easy to determine the relationship between specific working postures and depression. Some have reported that posture type did not show any relationship with mental processes [28]. Our cross-sectional study cannot show the causal associations between working posture related variables and depression. Thus, longitudinal studies should be conducted to elucidate these associations. It is necessary to conduct further studies in consideration of the various factors related to working posture, rather than studying the relationship between depression and working posture in which the demographic and environmental factors of workers are not considered. Furthermore, the variables from a single question were limited in detailed analysis and evaluation, and advanced measurement scales are required.

## 5. Conclusions

This study demonstrated that working posture is associated with workers’ depression. Taking the previous studies on this topic and this study together, it seems that working postures causing musculoskeletal pain, improper working postures maintained for a long time, and occupation type are associated with workers’ depression, rather than that a specific working posture induces depression. Therefore, appropriate management and intervention is required for pain-inducing or improper working postures in the workplace.

## Figures and Tables

**Table 1:** General characteristics of workers (*n* = 49,877).

Variables			Depression		
			No	Yes	*p*
Age	≤29		4344 (9.0)	76 (5.5)	<0.001
	30–49		19,741 (40.7)	427 (30.9)	
	50–64		17,073 (35.2)	525 (38.0)	
	≥65		7338 (15.1)	353 (25.6)	
Sex	Male		23,026 (47.5)	535 (38.7)	<0.001
	Female		25,470 (52.5)	846 (61.3)	
Education	≤Elementary school		4889 (10.1)	292 (21.1)	<0.001
	Middle–high school		22,891 (47.2)	660 (47.8)	
	≥College		20,716 (42.7)	429 (31.1)	
Occupation	WC worker		4500 (9.3)	106 (7.7)	<0.001
	BC worker		8818 (18.2)	158 (11.4)	
	PC worker		18,624 (38.4)	541 (39.2)	
	AL worker		16,554 (34.1)	576 (41.7)	
Shift work	No		44,392 (91.5)	1256 (90.9)	0.435
	Yes		4104 (8.5)	125 (9.1)	
Job satisfaction (1–4)			2.78 ± 0.55	2.38 ± 0.70	<0.001
Physical health	Back pain	No	41,754 (86.1)	758 (54.9)	<0.001
problem		Yes	67,42 (13.9)	623 (45.1)	
	Neck, shoulder, and arm pain	No	35,410 (73.0)	463 (33.5)	<0.001
		Yes	13,086 (27.0)	918 (66.5)	
	Hip, leg, knee, and foot pain	No	39,025 (80.5)	586 (42.4)	<0.001
		Yes	9471 (19.5)	795 (57.6)	
Working posture	Tiring or painful positions		3.08 ± 1.67	4.00 ± 1.86	<0.001
	Lifting or moving people		1.74 ± 0.97	1.80 ± 1.16	0.076
	Carrying or moving heavy loads		2.49 ± 1.33	2.84 ± 1.48	<0.001
	Standing		3.75 ± 1.69	3.87 ± 1.70	0.008
	Sitting		3.97 ± 1.64	3.99 ± 1.56	0.663
	Repetitive hand or arm movements		4.11 ± 1.92	4.52 ± 1.82	<0.001

**Table 2:** Working posture affecting depression in workers.

Variables		Total	Male	Female
		Odds Ratio (*p*)		
Working posture	Tiring or painful positions	**1.096 (<0.001)**	**1.098 (0.006)**	**1.095 (<0.001)**
	Lifting or moving people	1.041 (0.142)	**1.101 (0.025)**	1.005 (0.880)
	Carrying or moving heavy loads	0.991 (0.712)	0.939 (0.093)	1.041 (0.195)
	Standing	**0.938 (0.004)**	0.934 (0.056)	**0.938 (0.028)**
	Sitting	**1.049 (0.034)**	1.010 (0.782)	**1.068 (0.026)**
	Repetitive hand or arm movements	0.992 (0.644)	0.964 (0.207)	1.007 (0.758)
Age	≤29	1	1	1
	30–49	0.963 (0.770)	1.065 (0.753)	0.868 (0.407)
	50–64	1.007 (0.959)	1.007 (0.972)	0.941 (0.736)
	≥65	1.073 (0.650)	1.226 (0.385)	0.924 (0.711)
Education	≤Elementary school	1	1	1
	Middle–high school	0.902 (0.302)	0.892 (0.503)	0.993 (0.960)
	≥College	1.110 (0.410)	1.166 (0.457)	1.213 (0.267)
Occupation	WC worker	1	1	1
	BC worker	**0.755 (0.033)**	**0.613 (0.007)**	0.877 (0.493)
	PC worker	0.888 (0.323)	**0.651 (0.014)**	1.108 (0.548)
	AL worker	**0.549 (<0.001)**	**0.393 (<0.001)**	0.773 (0.176)
Shift work	No	1	1	1
	Yes	1.124 (0.246)	**1.383 (0.023)**	0.959 (0.773)
Job satisfaction (1–4)		**0.460 (<0.001)**	**0.424 (<0.001)**	**0.480 (<0.001)**
Physical health problem	Back pain	**2.035 (<0.001)**	**1.879 (<0.001)**	**2.130 (<0.001)**
	Neck, shoulder, and arm pain	**2.164 (<0.001)**	**2.312 (<0.001)**	**2.079 (<0.001)**
	Hip, leg, knee, and foot pain	**2.344 (<0.001)**	**2.756 (<0.001)**	**2.102 (<0.001)**

## Data Availability

All data used were from the 5th Korean Working Conditions Survey conducted by the Korea Occupational Safety and Health Agency in 2017.
